# Supplementary material for: Coumarin-Based Sulfonamide Derivatives as Potential DPP-IV Inhibitors: Pre-ADME Analysis, Toxicity Profile, Computational Analysis, and In Vitro Enzyme Assay
Source: Molecules. 2023 Jan 19;28(3):1004. doi: 10.3390/molecules28031004 (PMC9921777; doi:10.3390/molecules28031004)
Supplement: Supplementary file 1 [file molecules-28-01004-s001.zip › molecules-2062746-supplementary.pdf]

Article

Coumarin-Based Sulfonamide Derivatives as Potential DPP-IV Inhibitors: Pre-ADME Analysis, Toxicity Profile, Computational Analysis, and In Vitro Enzyme Assay

Pallavi Kishor Vawhal [1,*], Shailaja B. Jadhav [1], Sumit Kaushik [2], Kahnu Charan Panigrahi [3], Chandan Nayak [4], Humaira Urmee [5], Sharuk L. Khan [6], Falak A. Siddiqui [6], Fahadul Islam [7], Aziz Eftekhari [8], Abdullah R. Alzahrani [9], Mohd Fahami Nur Azlina [10], Md. Moklesur Rahman Sarker [11,12,*] and Ibrahim Abdel Aziz Ibrahim [9,*]

1 Department of Pharmaceutical Chemistry, PEA's Modern College of Pharmacy, Sector 21, Yamunanagar, Nigdi 411044, India
2 Faculty of Pharmacy, Raja Balwant Singh Engineering Technical Campus, Bichpuri, Agra 283105, India
3 Department of Pharmaceutics, Gayatri Institute of Science and Technology, Gyan Vihar, Gunupur 765022, India
4 Department of Pharmaceutics, School of Pharmaceutical Education and Research, Berhampur University, Ganjam 760007, India
5 Department of Pharmaceutical Science, North South University, Dhaka 1229, Bangladesh
6 Department of Pharmaceutical Chemistry, N.B.S. Institute of Pharmacy, Ausa 413520, India
7 Department of Pharmacy, Faculty of Allied Health Sciences, Daffodil International University, Dhaka 1207, Bangladesh
8 Research Center for Pharmaceutical Nanotechnology, Biomedicine Institute, Tabriz University of Medical Sciences, Tabriz 51665118, Iran
9 Department of Pharmacology and Toxicology, Faculty of Medicine, Umm Al-Qura University, Makkah 4089-6928, Saudi Arabia
10 Department of Pharmacology, Faculty of Medicine, University Kebangsaan Malaysia, Jalan Yacob Latif, Kuala Lumpur 56000, Malaysia
11 Department of Pharmacy, State University of Bangladesh, 77 Satmasjid Road, Dhanmondi, Dhaka 1205, Bangladesh
12 Health Med Science Research Network, 3/1, Block F, Lalmatia, Dhaka 1207, Bangladesh
* Correspondence: vawhalpallavi@gmail.com (P.K.V.); moklesur2002@yahoo.com (M.M.R.S.); iamustafa@uqu.edu.sa (I.A.A.I.)

Supplementary File:

Table S1. Calculations of oral bioavailability parameters of the designed derivatives.

Molecules Codes	Ro5					Veber's rule	
	Log *P*	Mol. Wt.	HBA	HBD	*V**	TPSA (Å²)	No. of rotatable bonds
NL	1.42	315.29	6	2	0	75.43	5
6a	2.85	335.76	4	1	0	84.76	1
6b	2.26	336.75	5	1	0	97.65	1
6c	2.12	336.75	5	1	0	97.65	1
6d	2.12	336.75	5	1	0	97.65	1
6e	2.36	342.78	5	1	0	125.89	1
6f	2.32	384.81	6	1	0	142.96	1
6g	2.89	341.79	4	1	0	113	2
6h	1.69	325.73	5	2	0	113.44	2
6i	2.55	375.79	5	2	0	113.44	2
6j	2.96	389.81	5	2	0	113.44	1
6k	1.64	273.69	5	1	0	84.76	1
6l	1.96	287.72	5	1	0	84.76	1
6m	2.28	301.75	5	1	0	84.76	1
6n	2.24	301.75	5	1	0	84.76	2
6o	2.45	351.76	5	2	0	104.99	2
6p	2.44	351.76	5	2	0	104.99	2
6q	2.44	351.76	5	2	0	104.99	1
6r	3.17	349.79	4	1	0	84.76	1
6s	3.18	349.79	4	1	0	84.76	1
6t	2.89	349.79	5	1	0	84.76	1

Table S2. Pre-ADME characteristics of generated compounds.

Codes	Pharmacokinetics									Drug-likeness			
	GI abs.	BBB pen.	P-gp sub.	CYP1A2 inhibitors	CYP2C19 inhibitors	CYP2C9 inhibitors	CYP2D6 inhibitors	CYP3A4 inhibitors	Log K_p (skin permeation, cm/s)	Ghose	Egan	Muegge	Bioavailability Score
NL	H	N	Y	N	N	N	Y	N	-8.11	0	0	0	0.55
6a	H	N	N	Y	Y	Y	N	N	-6.03	0	0	0	0.55
6b	H	N	N	Y	N	N	N	N	-6.56	0	0	0	0.55
6c	H	N	N	Y	N	N	N	N	-6.79	0	0	0	0.55
6d	H	N	N	Y	N	N	N	N	-6.79	0	0	0	0.55
6e	L	N	N	Y	Y	Y	N	Y	-6.52	0	0	0	0.55
6f	H	N	N	N	Y	Y	N	N	-6.77	0	1	0	0.55
6g	H	N	N	Y	Y	Y	N	N	-6.06	0	0	0	0.55
6h	H	N	N	N	N	N	N	N	-7.05	0	0	0	0.55
6i	H	N	N	N	Y	N	N	N	-6.34	0	0	0	0.55
6j	H	N	N	Y	Y	Y	N	N	-6.17	0	0	0	0.55
6k	H	N	N	N	N	N	N	N	-6.76	0	0	0	0.55
6l	H	N	N	N	N	N	N	N	-6.58	0	0	0	0.55
6m	H	N	N	Y	Y	N	N	N	-6.29	0	0	0	0.55
6n	H	N	N	Y	N	N	N	N	-6.36	0	0	0	0.55
6o	H	N	N	N	N	Y	N	N	-6.38	0	0	0	0.55
6p	H	N	N	N	N	N	N	N	-6.38	0	0	0	0.55
6q	H	N	N	N	N	Y	N	N	-6.38	0	0	0	0.55

6r	H	N	N	Y	Y	Y	N	N	-5.86	0	0	0	0.55
6s	H	N	N	Y	Y	Y	N	N	-5.86	0	0	0	0.55
6t	H	N	N	Y	Y	Y	N	N	-6.15	0	0	0	0.55

Table S3. Pre-Tox profile of designed molecules.

<table>
<tr><th rowspan="2">Compound codes</th><th colspan="8">Parameters</th></tr>
<tr><th>LD50 (mg/kg)</th><th>Toxicity class</th><th>Prediction accuracy (%)</th><th>Hepatotoxicity (Probability)</th><th>Carcinogenicity (Probability)</th><th>Immunotoxicity (Probability)</th><th>Mutagenicity (Probability)</th><th>Cytotoxicity (Probability)</th></tr>
<tr><td>NL</td><td>210</td><td>3</td><td>68.07</td><td>I (0.89)</td><td>I (0.65)</td><td>I (0.97)</td><td>I (0.77)</td><td>I (0.77)</td></tr>
<tr><td>6a</td><td>2100</td><td>5</td><td rowspan="2">67.38</td><td rowspan="2">A (0.50)</td><td rowspan="2">I (0.66)</td><td rowspan="20">I (0.99)</td><td rowspan="2">I (0.76)</td><td rowspan="2">I (0.78)</td></tr>
<tr><td>6b</td><td>2100</td><td>5</td></tr>
<tr><td>6c</td><td>2500</td><td>5</td><td rowspan="2">54.26</td><td>A (0.58)</td><td>I (0.63)</td><td rowspan="2">I (0.81)</td><td>I (0.75)</td></tr>
<tr><td>6d</td><td>2500</td><td>5</td><td>A (0.53)</td><td>I (0.62)</td><td>I (0.78)</td></tr>
<tr><td>6e</td><td>4500</td><td>5</td><td>67.38</td><td rowspan="2">A (0.63)</td><td rowspan="2">I (0.63)</td><td>I (0.84)</td><td>I (0.80)</td></tr>
<tr><td>6f</td><td>4500</td><td>5</td><td rowspan="7">54.26</td><td>I (0.83)</td><td rowspan="2">I (0.78)</td></tr>
<tr><td>6g</td><td>2100</td><td>5</td><td>A (0.57)</td><td>I (0.66)</td><td>I (0.76)</td></tr>
<tr><td>6h</td><td>300</td><td>3</td><td rowspan="3">A (0.59)</td><td rowspan="3">I (0.59)</td><td rowspan="2">I (0.86)</td><td rowspan="2">I (0.81)</td></tr>
<tr><td>6i</td><td>1370</td><td>4</td></tr>
<tr><td>6j</td><td>25000</td><td>6</td><td>I (0.85)</td><td>I (0.82)</td></tr>
<tr><td>6k</td><td>2100</td><td>5</td><td>I (0.57)</td><td>I (0.68)</td><td>I (0.70)</td><td>I (0.74)</td></tr>
<tr><td>6l</td><td>890</td><td>4</td><td>I (0.59)</td><td>I (0.70)</td><td>I (0.68)</td><td>I (0.67)</td></tr>
<tr><td>6m</td><td>179</td><td>3</td><td rowspan="7">67.38</td><td>A (0.53)</td><td>I (0.60)</td><td rowspan="4">I (0.79)</td><td>I (0.75)</td></tr>
<tr><td>6n</td><td>179</td><td>3</td><td rowspan="4">A (0.51)</td><td rowspan="4">I (0.61)</td><td rowspan="3">I (0.78)</td></tr>
<tr><td>6o</td><td>179</td><td>3</td></tr>
<tr><td>6p</td><td>179</td><td>3</td></tr>
<tr><td>6q</td><td>179</td><td>3</td><td rowspan="3">I (0.77)</td><td>I (0.79)</td></tr>
<tr><td>6r</td><td>2100</td><td>5</td><td rowspan="2">A (0.52)</td><td rowspan="2">I (0.65)</td><td rowspan="2">I (0.80)</td></tr>
<tr><td>6s</td><td>2100</td><td>5</td></tr>
<tr><td>6t</td><td>7900</td><td>6</td><td>54.26</td><td>I (0.61)</td><td>I (0.69)</td><td>I (0.69)</td><td>I (0.67)</td></tr>
</table>

Where: I, Inactive; A, Active.

Table S4. The BOILED-Egg plots of designed molecules

Molecule	BOILED-Egg plot
6a	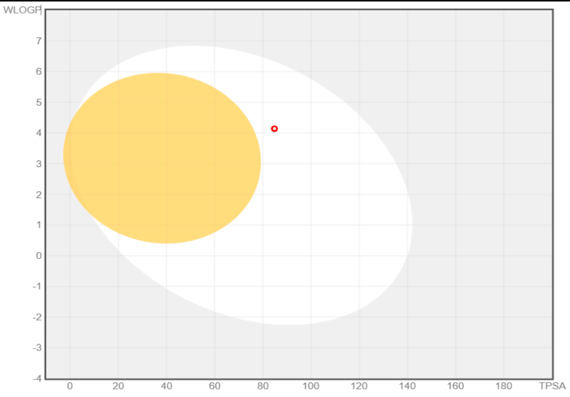

6b	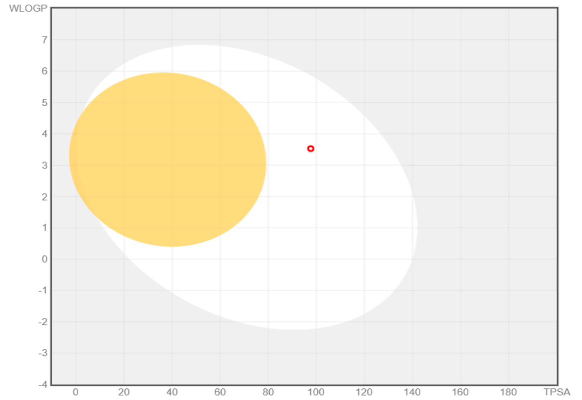 WLOGP 7 6 5 4 3 2 1 0 -1 -2 -3 -4 0 20 40 60 80 100 120 140 160 180 TPSA
6c	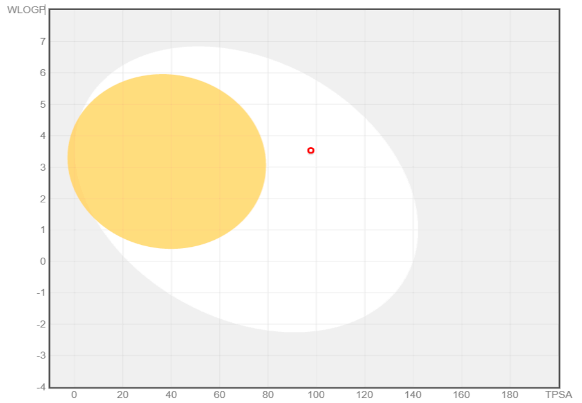 WLOGP 7 6 5 4 3 2 1 0 -1 -2 -3 -4 0 20 40 60 80 100 120 140 160 180 TPSA
6d	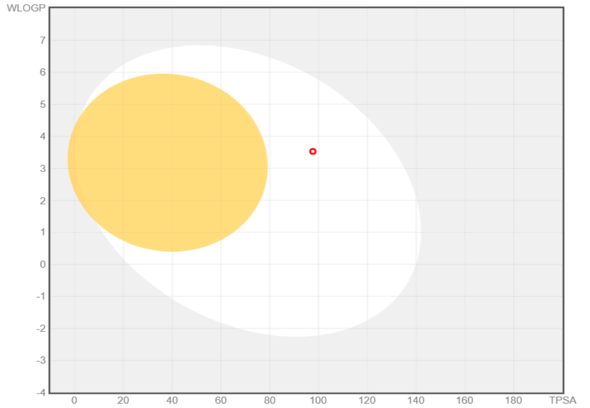 WLOGP 7 6 5 4 3 2 1 0 -1 -2 -3 -4 0 20 40 60 80 100 120 140 160 180 TPSA
6e	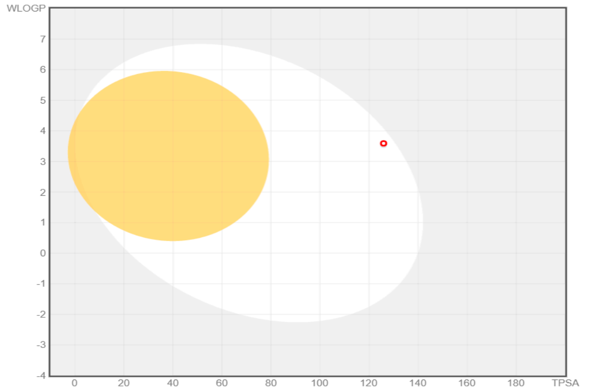 WLOGP 7 6 5 4 3 2 1 0 -1 -2 -3 -4 0 20 40 60 80 100 120 140 160 180 TPSA

6f	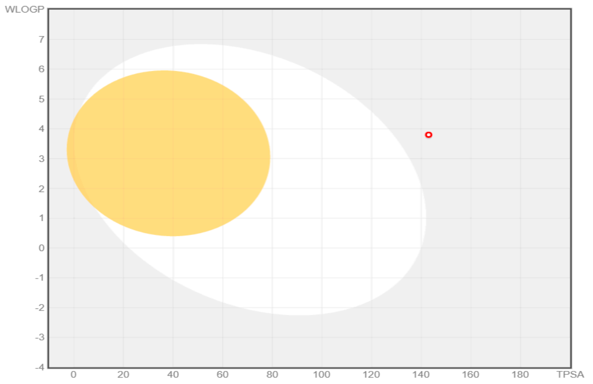WLOGP<br>7 6 5 4 3 2 1 0 -1 -2 -3 -4<br>0 20 40 60 80 100 120 140 160 180<br>TPSA
6g	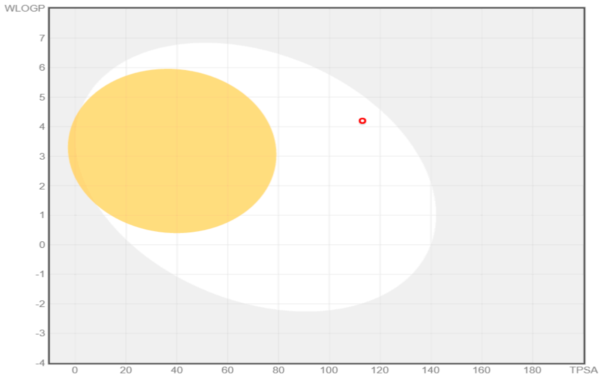WLOGP<br>7 6 5 4 3 2 1 0 -1 -2 -3 -4<br>0 20 40 60 80 100 120 140 160 180<br>TPSA
6h	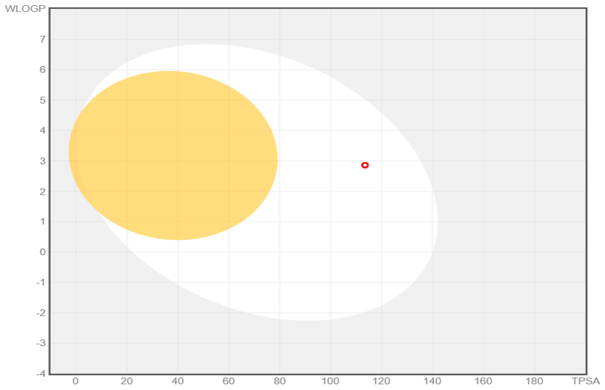WLOGP<br>7 6 5 4 3 2 1 0 -1 -2 -3 -4<br>0 20 40 60 80 100 120 140 160 180<br>TPSA
6i	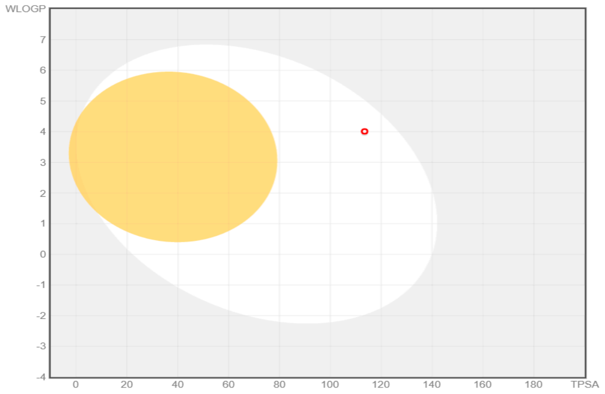WLOGP<br>7 6 5 4 3 2 1 0 -1 -2 -3 -4<br>0 20 40 60 80 100 120 140 160 180<br>TPSA

<table>
<tr><td>6j</td><td>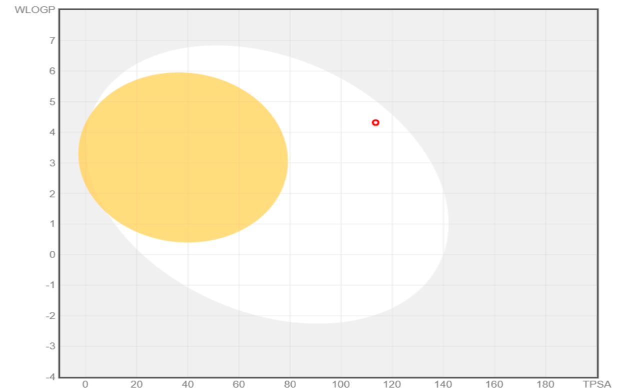
</td></tr>
<tr><td>6k</td><td>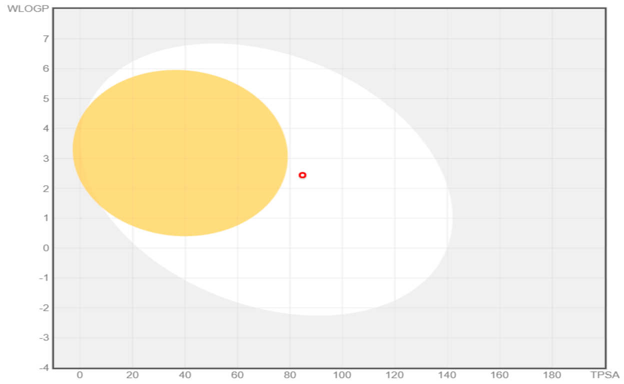
</td></tr>
<tr><td>6l</td><td>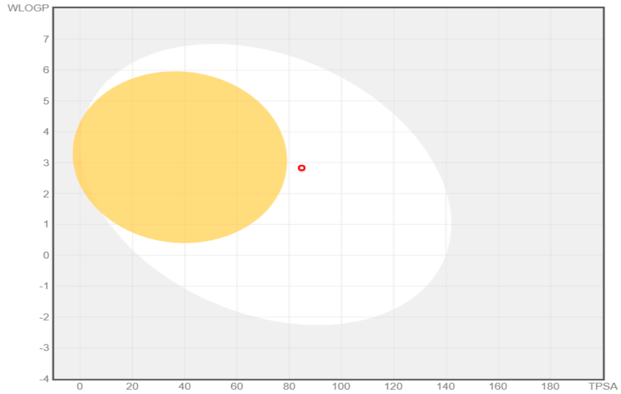
</td></tr>
<tr><td>6m</td><td>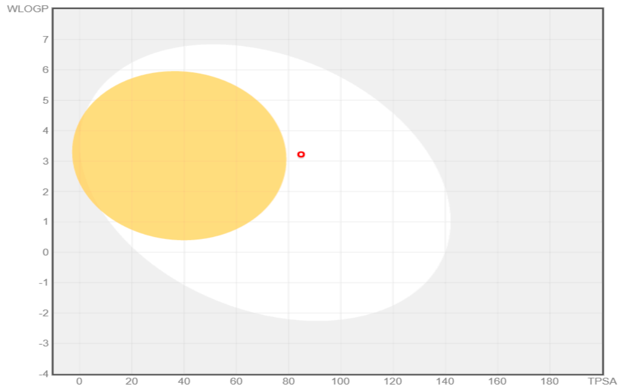
</td></tr>
</table>

6n	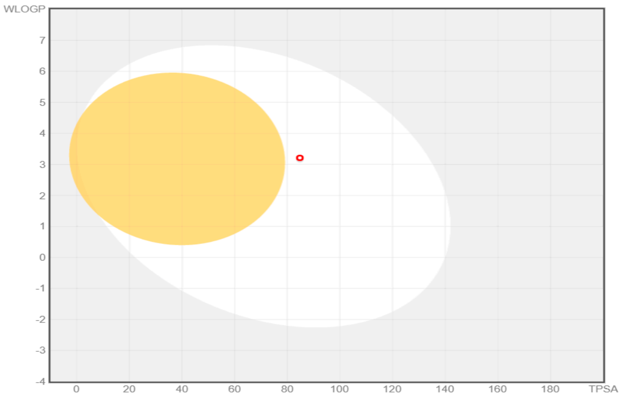 WLOGP 7 6 5 4 3 2 1 0 -1 -2 -3 -4 0 20 40 60 80 100 120 140 160 180 TPSA
6o	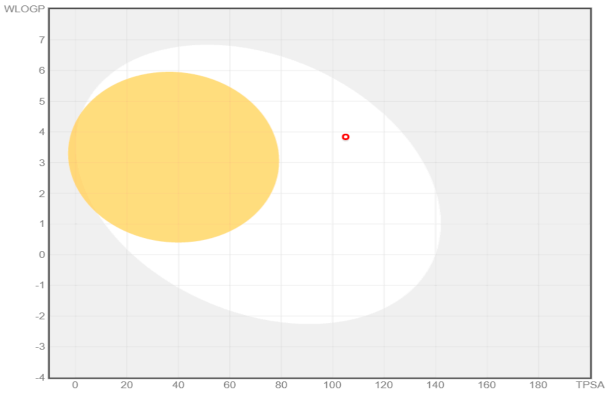 WLOGP 7 6 5 4 3 2 1 0 -1 -2 -3 -4 0 20 40 60 80 100 120 140 160 180 TPSA
6p	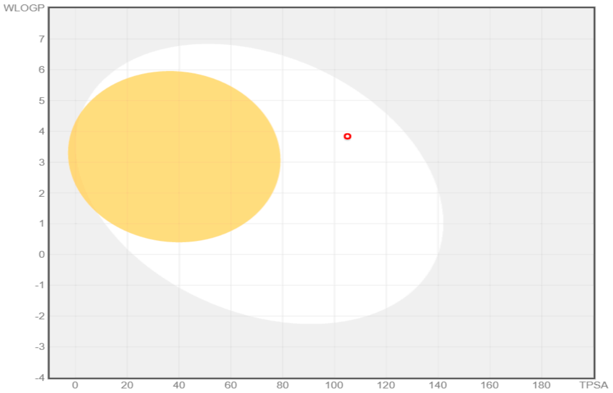 WLOGP 7 6 5 4 3 2 1 0 -1 -2 -3 -4 0 20 40 60 80 100 120 140 160 180 TPSA
6q	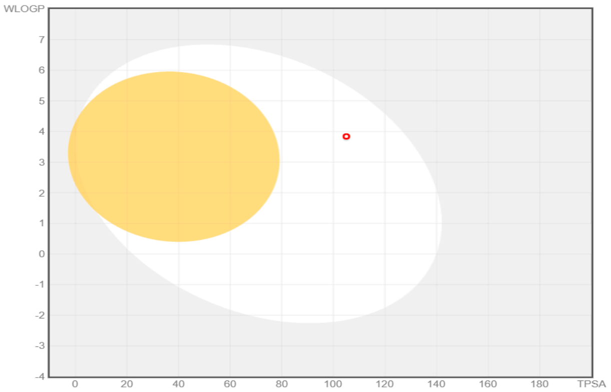 WLOGP 7 6 5 4 3 2 1 0 -1 -2 -3 -4 0 20 40 60 80 100 120 140 160 180 TPSA

6r	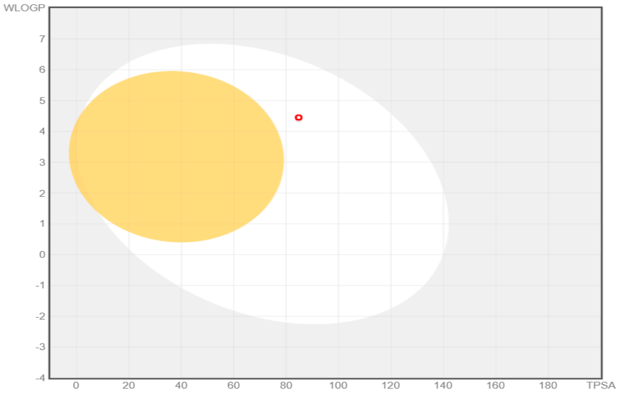 WLOGP<br>7 6 5 4 3 2 1 0 -1 -2 -3 -4<br>0 20 40 60 80 100 120 140 160 180 TPSA
6s	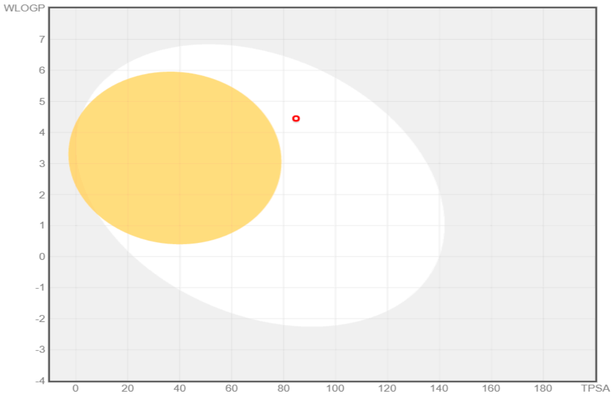 WLOGP<br>7 6 5 4 3 2 1 0 -1 -2 -3 -4<br>0 20 40 60 80 100 120 140 160 180 TPSA
6t	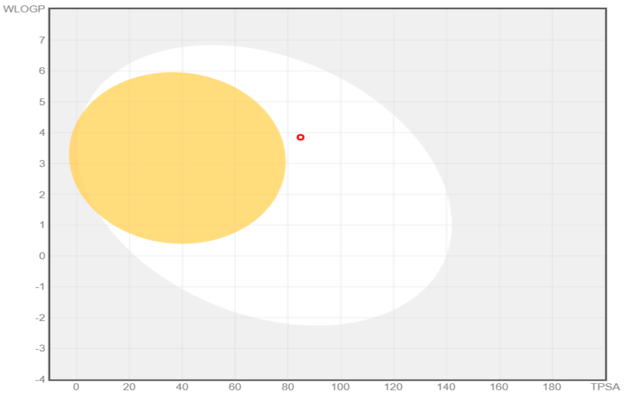 WLOGP<br>7 6 5 4 3 2 1 0 -1 -2 -3 -4<br>0 20 40 60 80 100 120 140 160 180 TPSA
NL	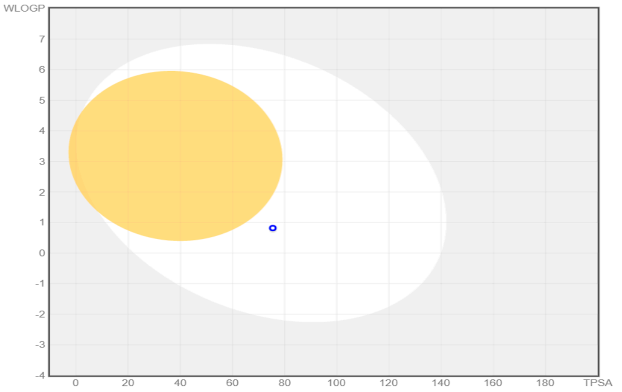 WLOGP<br>7 6 5 4 3 2 1 0 -1 -2 -3 -4<br>0 20 40 60 80 100 120 140 160 180 TPSA

Table S5. 3D-docking poses of molecules with active amino acid residues

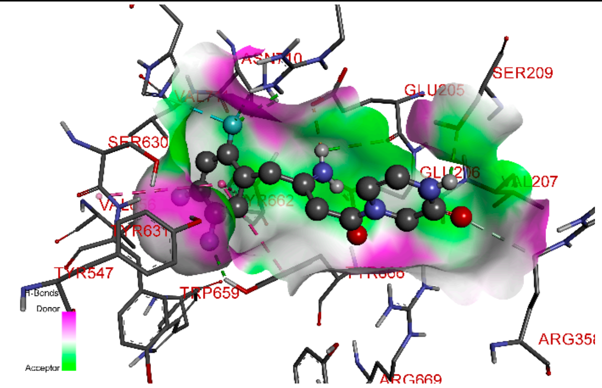

Reference inhibitor

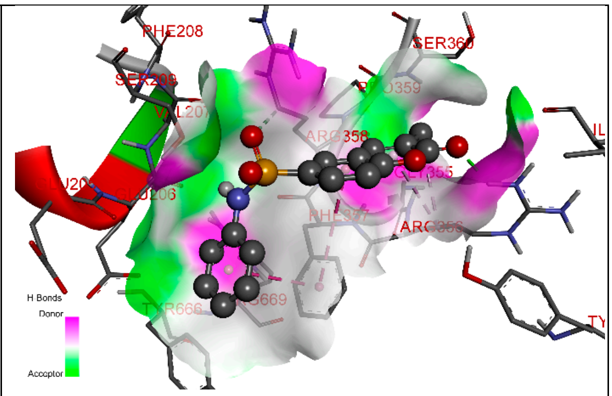

6a

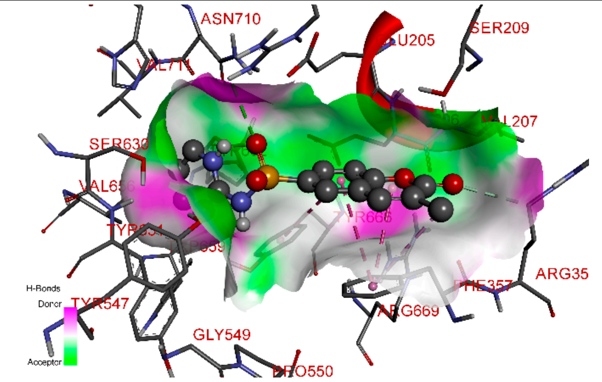

6b

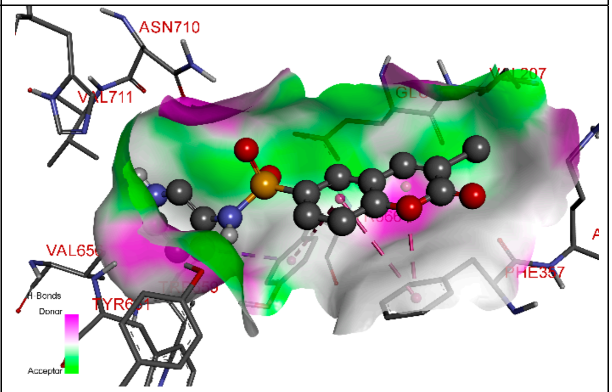

6c

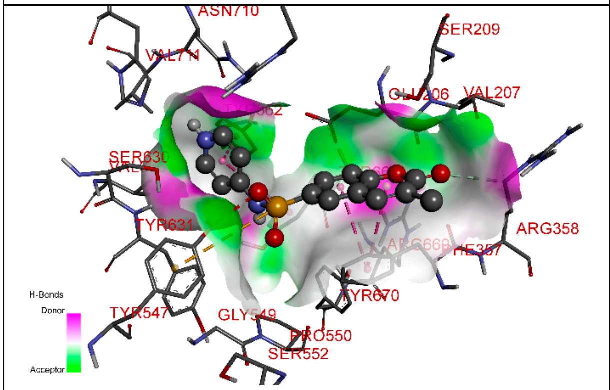

6d

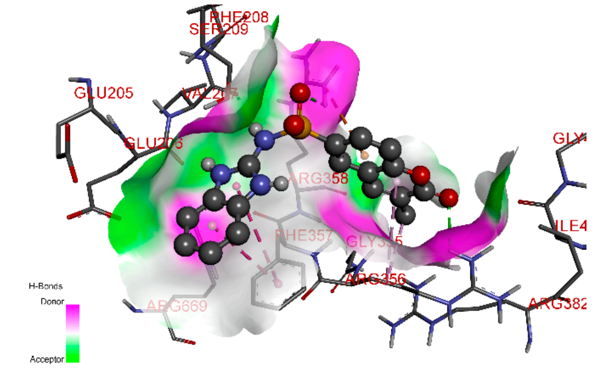

6i

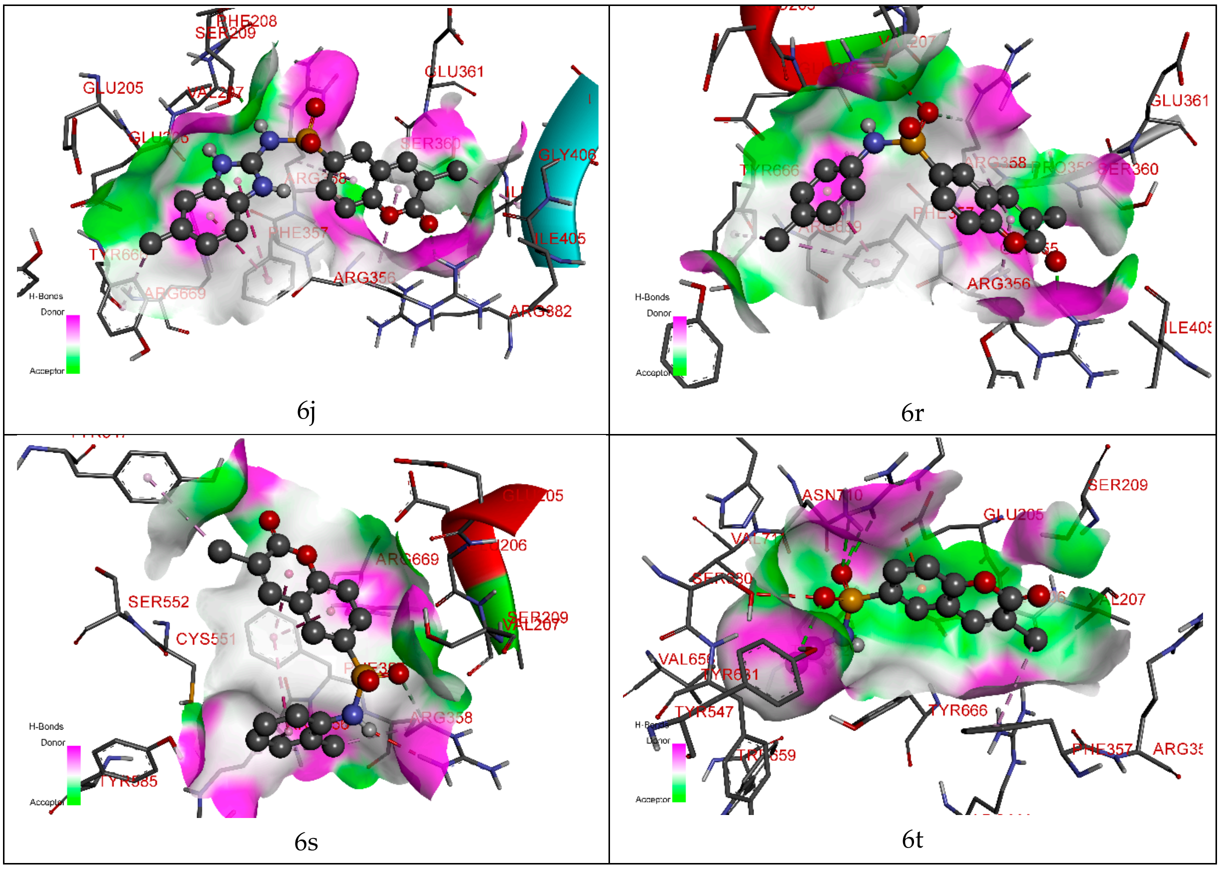

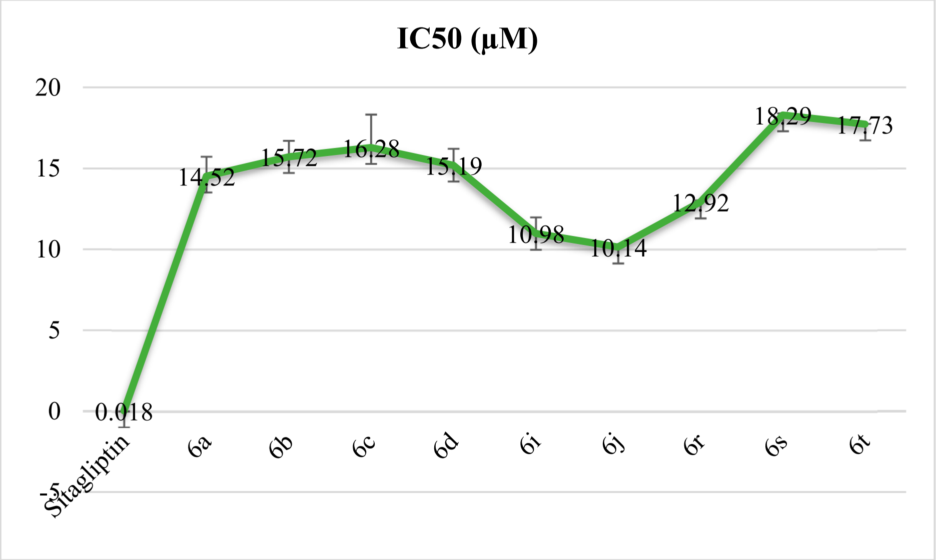

Figure S1. *In vitro* enzyme assay of synthesized compounds

Mass Spectrum of Synthesized Compounds:

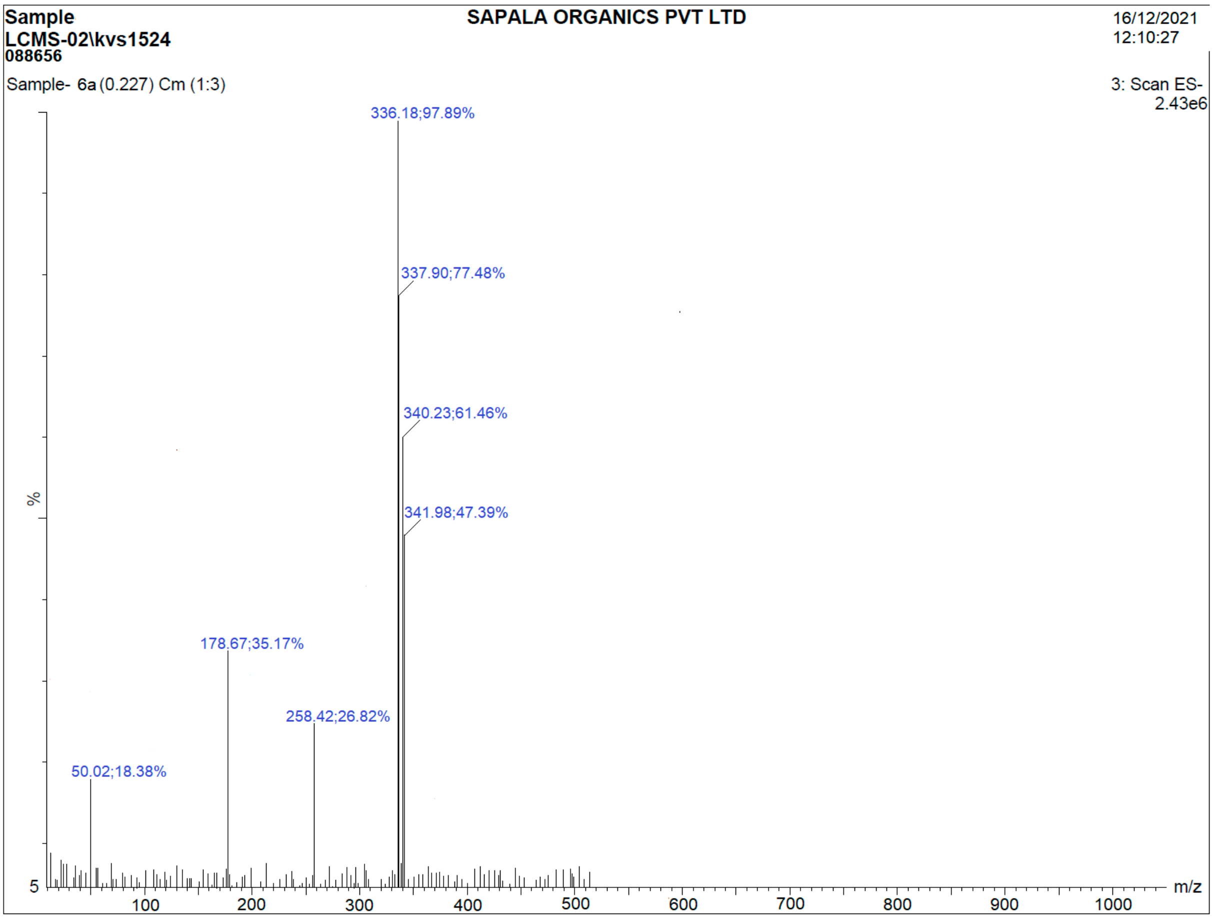

Figure S2. Mass spectra of compound 6a

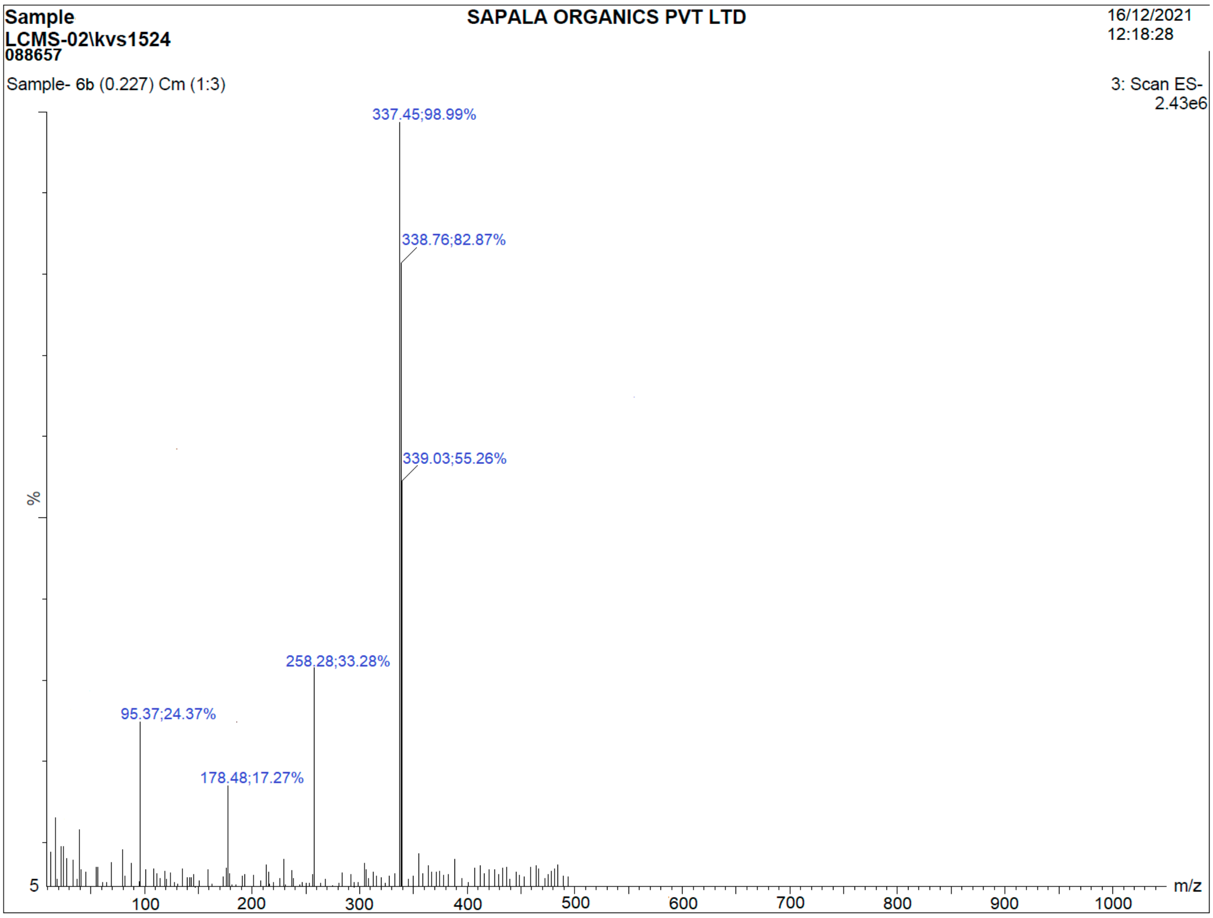

Figure S3. Mass spectra of compound 6b

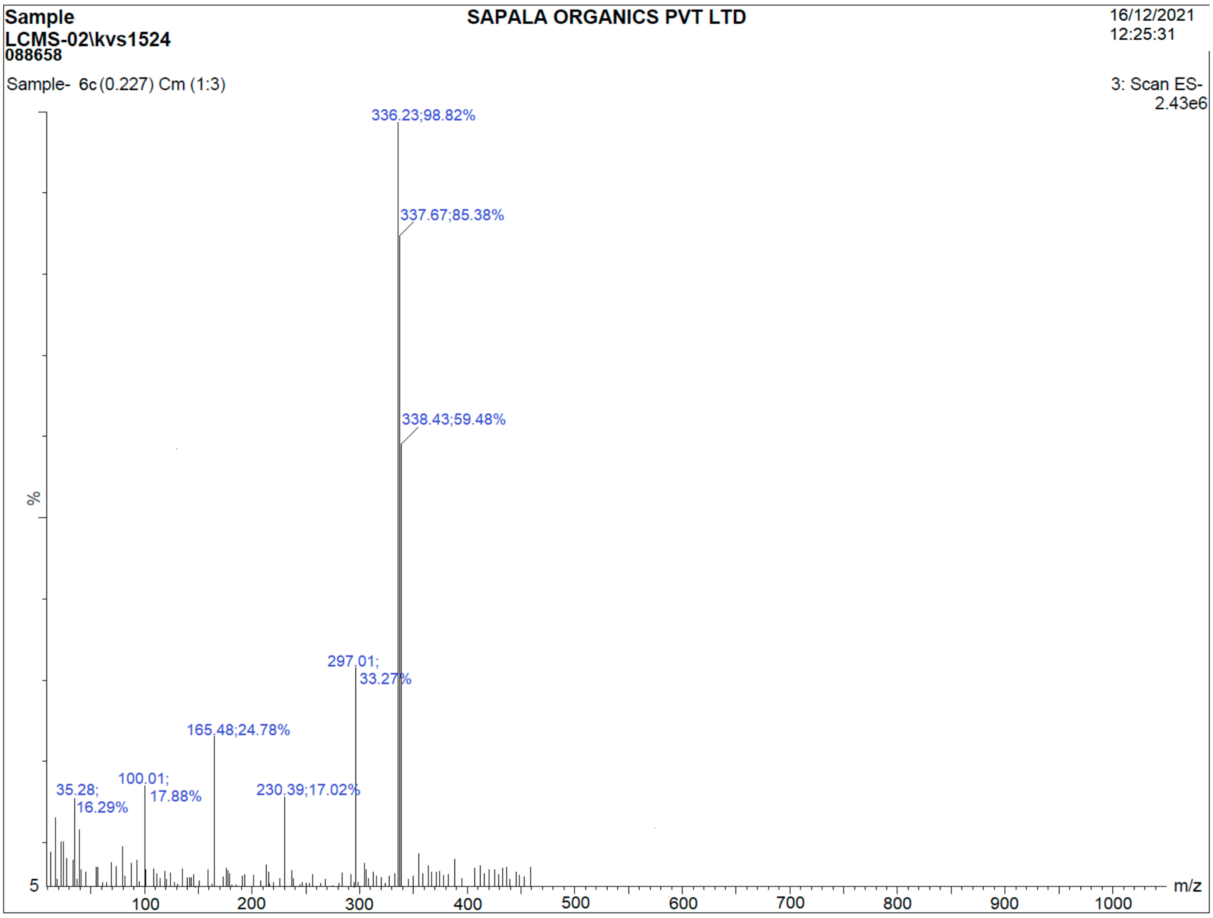

Figure S4. Mass spectra of compound 6c

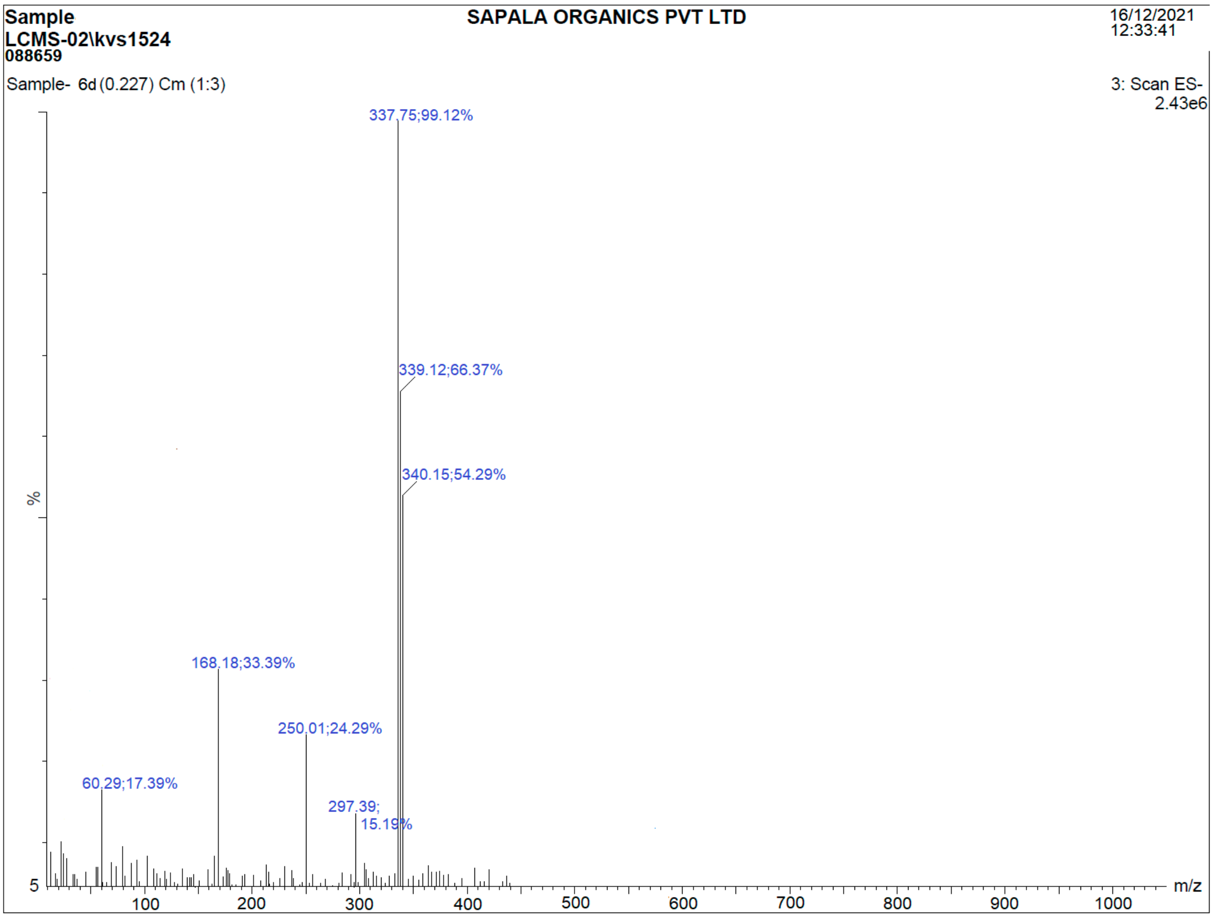

Figure S5. Mass spectra of compound 6d

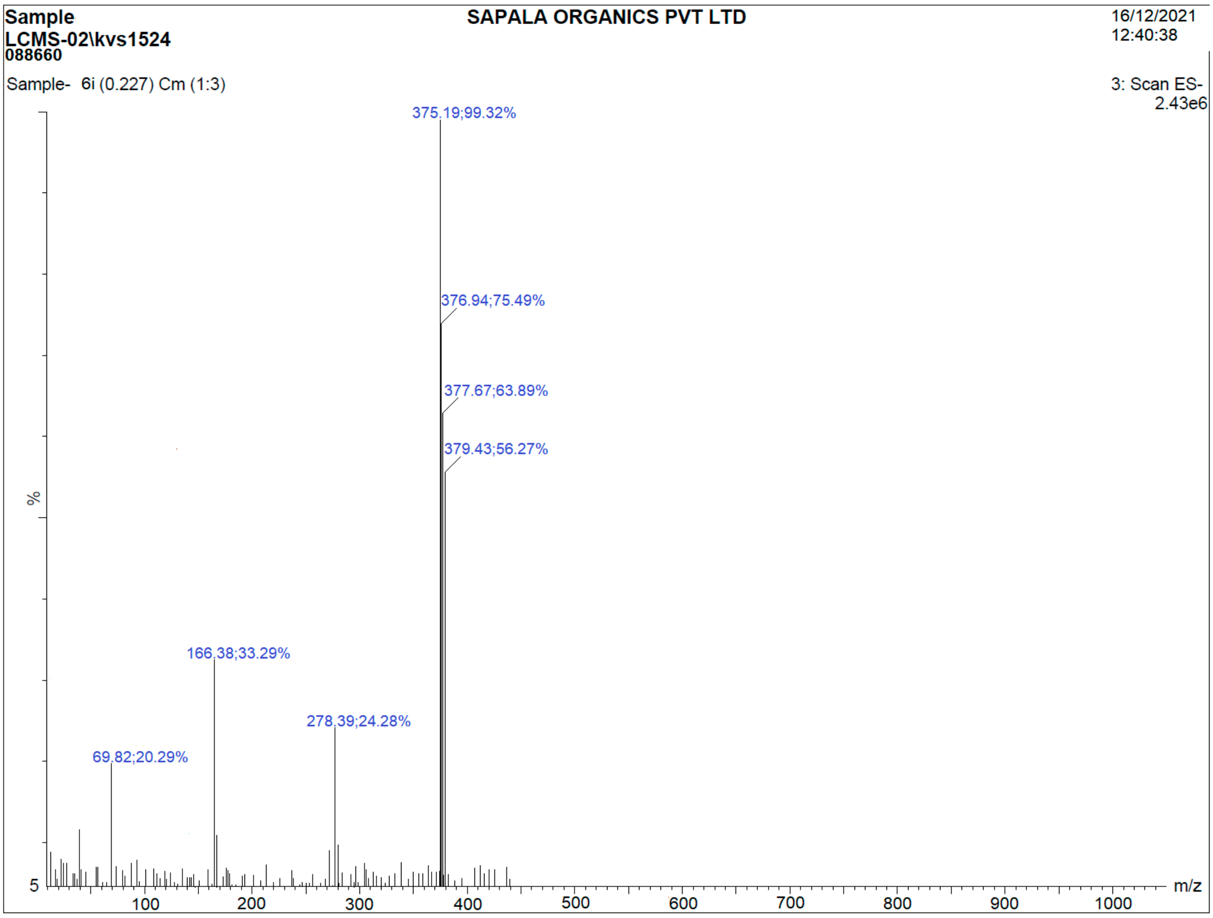

Figure S6. Mass spectra of compound 6i

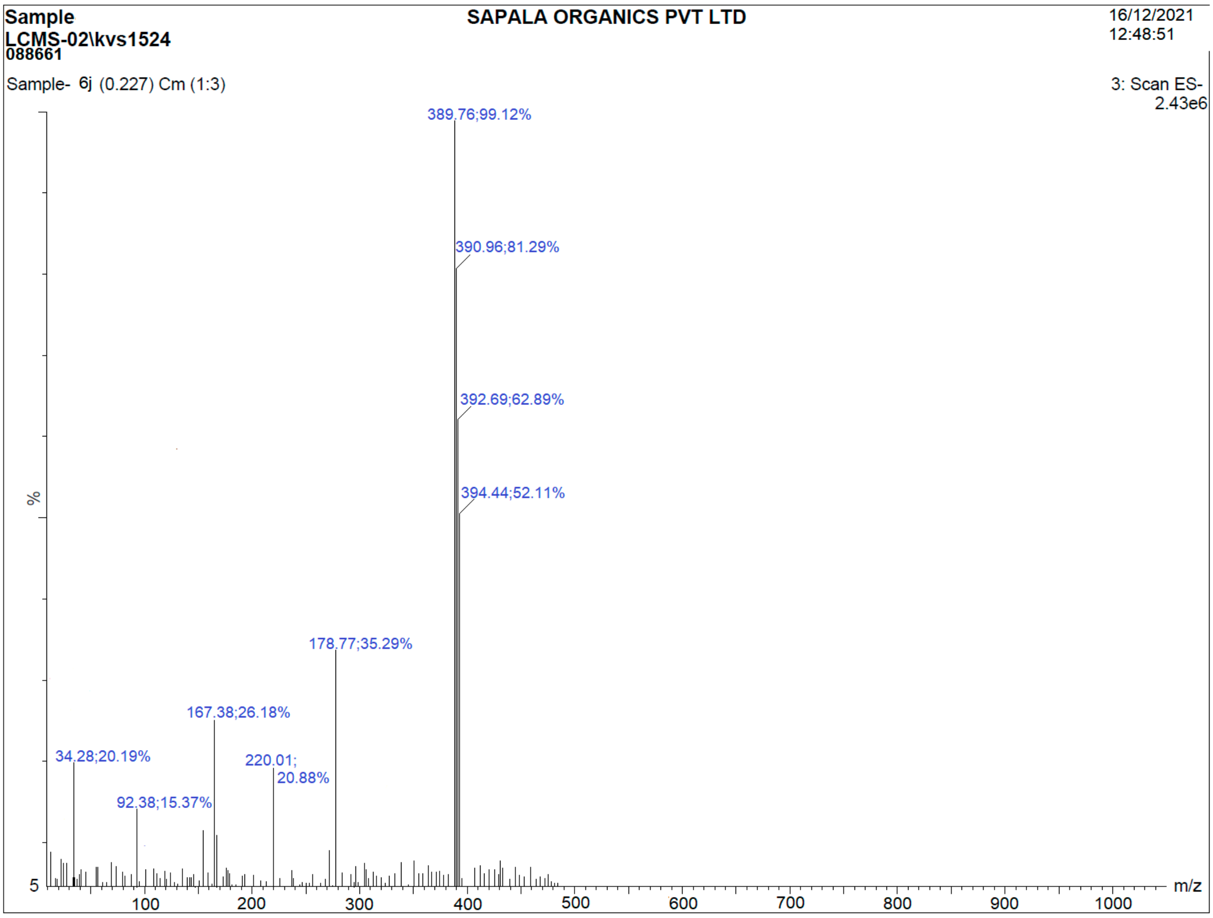

Figure S7. Mass spectra of compound 6j

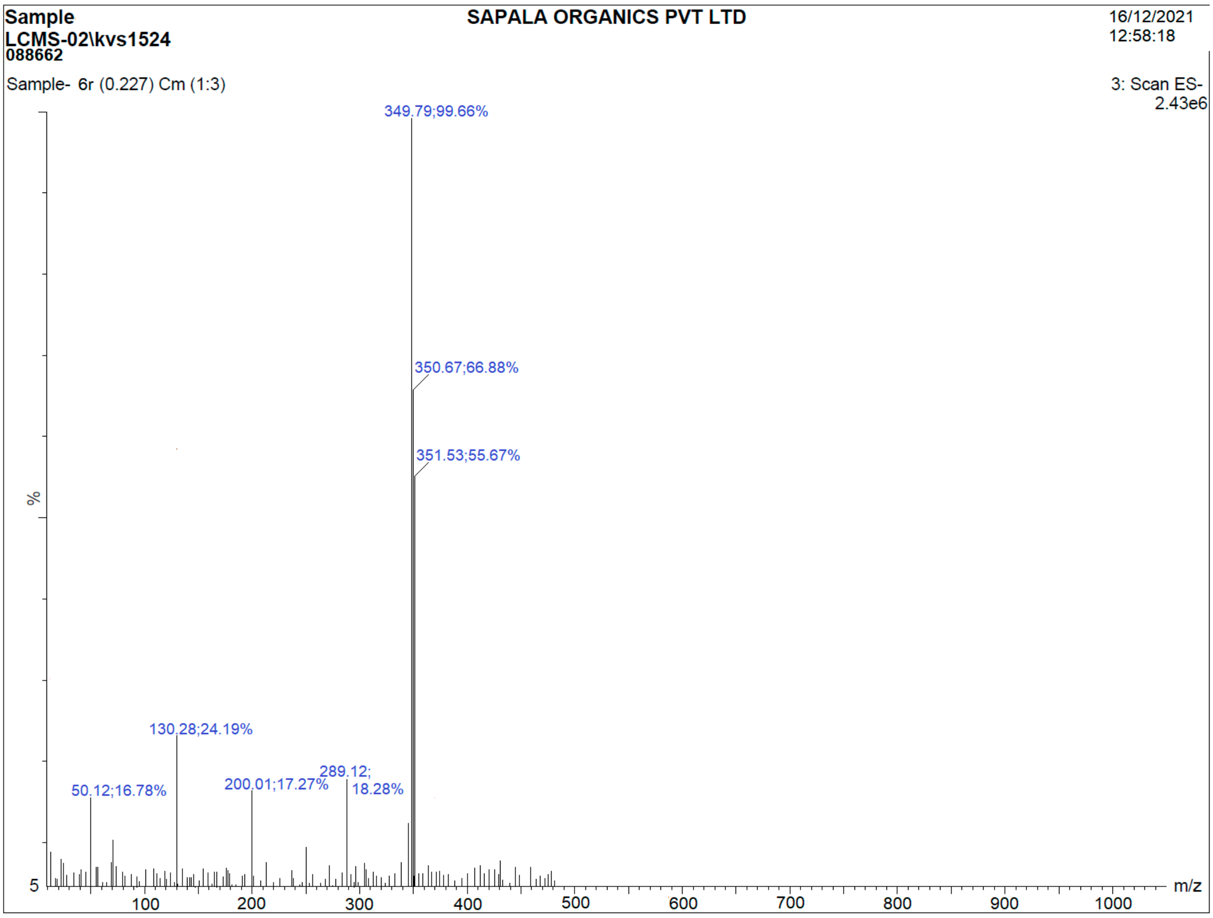

Figure S8. Mass spectra of compound 6r

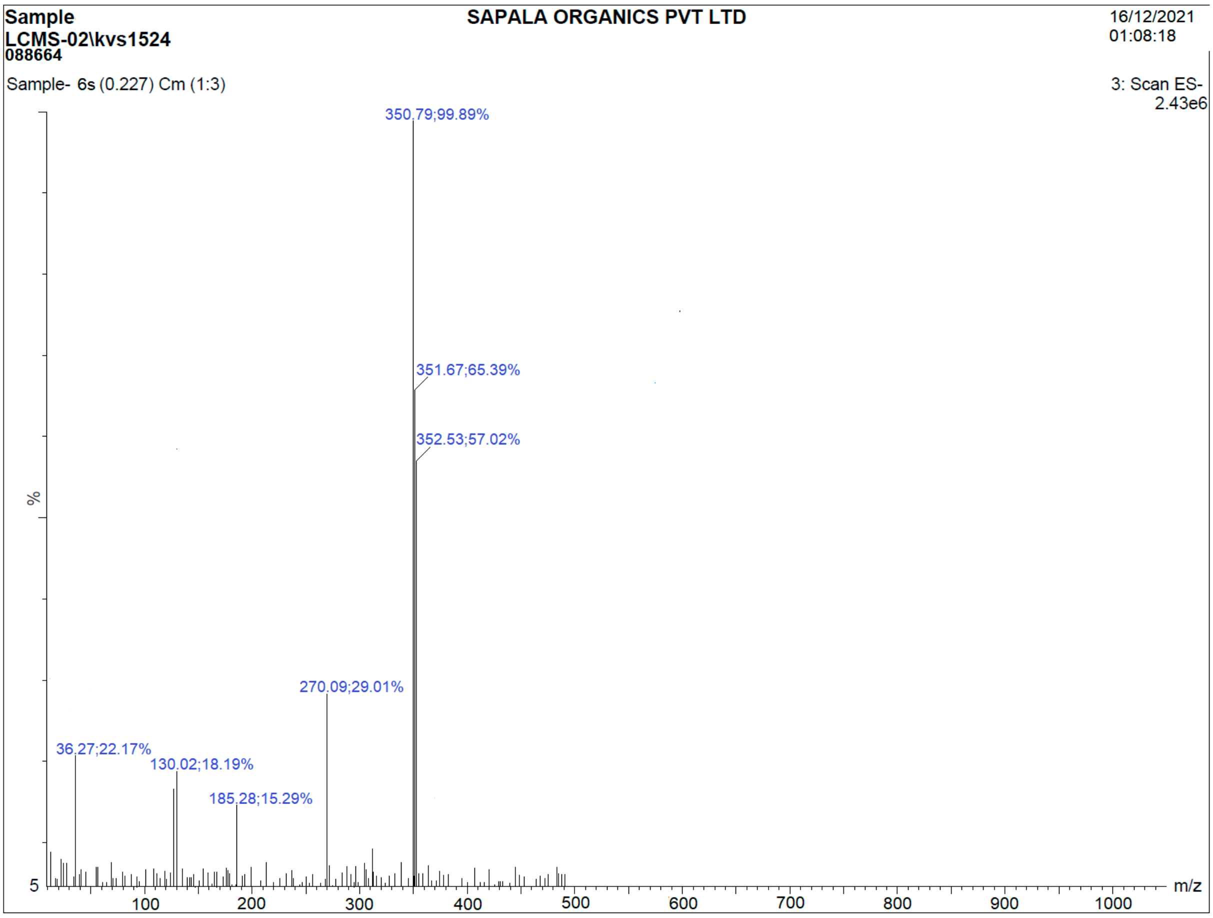

Figure S9. Mass spectra of compound 6s

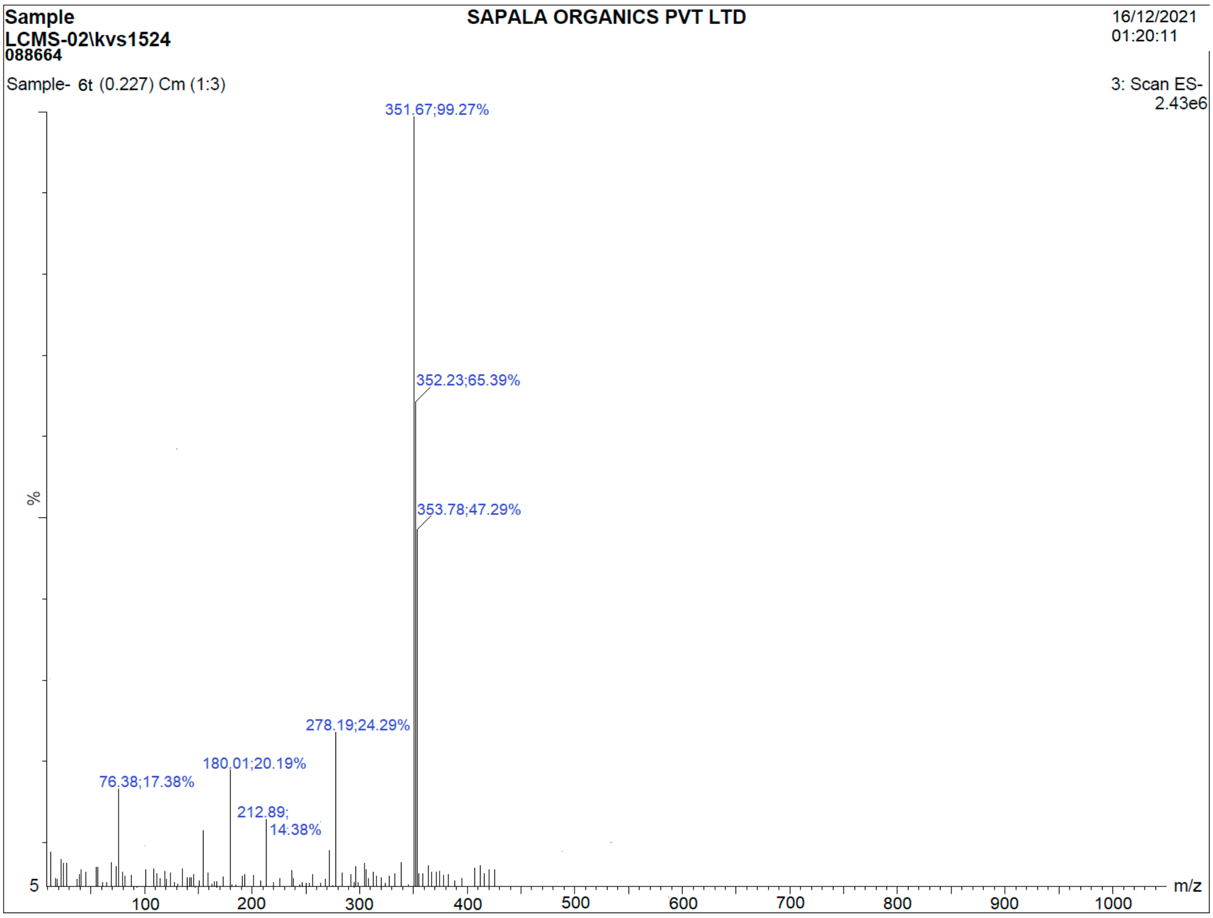

Figure S10. Mass spectra of compound 6t
